# Wealth and regional disparities in child undernutrition: insights from national demographic and health survey

**DOI:** 10.3389/fpubh.2025.1654403

**Published:** 2025-08-15

**Authors:** Lijing Tan, Muhammad Shahid, Jiayi Song, Hafiz Muhammad Naveed, Itbar Khan

**Affiliations:** ^1^School of Management, Shenzhen University of Information Technology, Shenzhen, China; ^2^College of Management, Shenzhen University, Shenzhen, China; ^3^School of Healthcare Management, Tsinghua University, Beijing, China; ^4^Business School, Xiamen Institute of Technology, Xiamen, China

**Keywords:** child undernutrition, socio-economic inequalities, wealth and regional disparities, concentration index, Oaxaca-Blinder decomposition

## Abstract

**Introduction:**

In developing countries like Pakistan, the prevalence of malnutrition embodies a multifaceted development challenge, intricately linked to structural inequalities, with disproportionate burdens among socioeconomically and geographically disadvantaged populations.

**Methods:**

Drawing on the most recent Pakistan Demographic and Health Survey (2017–18), this study examines the magnitude of child undernutrition disparities across wealth quintiles and geographic regions. This study employs the Concentration Index (CI) with decomposition analysis, alongside Oaxaca-Blinder decomposition as robust.

**Results:**

The results of the concentration index reveal that child undernutrition in Pakistan is deeply rooted in socioeconomic disparities, with household wealth contributing the largest share (45.6%) to overall inequality. The negative values of both relative and absolute CI confirm a pronounced pro-poor concentration of malnutrition. Regional disparities also play a significant role, with Sindh, Khyber Pakhtunkhwa, Balochistan, and FATA jointly accounting for 12.9% of inequality. Notably, maternal illiteracy emerges as a critical determinant, explaining 24.1% of the observed nutritional inequity. The results of the OaxacaBlinder decomposition disclose a pronounced wealth gap in child malnutrition, with poor households experiencing a 25.5 percentage point higher likelihood of malnutrition compared to their wealthier counterparts. Approximately 65% of the explained disparity is attributed to household wealth status, maternal education, and geographic region, underscoring the structural nature of nutritional inequalities in Pakistan.

**Conclusion:**

The study concludes that child malnutrition in Pakistan is fundamentally a structural equity issue. Addressing this issue requires multisectoral policy interventions focused on economic empowerment, regional development, and girls’ education to break the intergenerational cycle of undernutrition. Moreover, Pakistan’s extreme concentration of wealth and regional marginalization create distinct disparities that standard regional models do not fully capture. The dominance of wealth and regional factors, accounting for 65% of the explained gap, highlights the need for structural solutions, such as wealth redistribution and provincial equity funds, rather than isolated health interventions.

## 1 Introduction

Child undernutrition remains a critical global public health challenge with far-reaching consequences for human development and social equity. According to recent UNICEF estimates, approximately 150.2 million children under five suffer from stunting, while 42.8 million experiences wasting—including 12.2 million with severe wasting. Simultaneously, childhood overweight affects another 35.5 million, highlighting the escalating dual burden of malnutrition (1). These conditions not only impair physical growth and cognitive potential but also reinforce cycles of poverty and reduced economic productivity, demanding urgent policy attention (2). Socioeconomic disparities exacerbate these challenges, as children from the poorest households face 2–3 times higher risks of malnutrition compared to their wealthier peers (3, 4). Such inequities persist globally but are most severe in South Asia and sub-Saharan Africa, where structural barriers—including wealth gaps, regional marginalization, and limited education—sustain nutritional disadvantages (5, 6). The long-term implications are profound: early childhood undernutrition undermines cognitive development, lowers educational attainment, and perpetuates intergenerational poverty cycles (7, 8).

Within this global context, Pakistan represents a particularly concerning case. With 38% of children under five experiencing stunting (PDHS 2018), Pakistan’s malnutrition burden exceeds both regional and global averages (9, 10). The national prevalence obscures severe inequalities: children in the poorest wealth quintile show 57% stunting rates compared to just 28% among the wealthiest, while rural children consistently fare worse than their urban counterparts (11–13). These disparities reflect Pakistan’s complex landscape of structural inequities, where economic disadvantage intersects with geographic marginalization and limited educational opportunities (14, 15).

The roots of these nutritional inequalities are well-documented yet persistent. Poverty constrains access to diverse, nutrient-rich foods and essential health services (16, 17), while low maternal education limits nutrition knowledge and caregiving practices (18, 19). Geographic factors compound these challenges, with conflict-affected regions like Balochistan and FATA showing significantly worse outcomes than more developed areas (20, 21). Compared to its South Asian neighbors, Pakistan has made slower progress in reducing these disparities, suggesting unique structural barriers to nutritional equity (22, 23).

Existing research has established several critical gaps in understanding Pakistan’s malnutrition inequalities. First, most studies examine stunting, wasting, or underweight separately (22, 24, 25), missing the overlapping nature of nutritional deprivation captured by the Composite Index of Anthropometric Failure (CIAF). Second, while socioeconomic gradients are well-documented (3, 4), few studies quantify the exact contributions of wealth and regional disparities. Third, provincial-level inequalities remain underexplored despite stark variations in infrastructure and service access (5, 6).

This study advances the current literature by applying rigorous methodological techniques to examine multidimensional malnutrition disparities in Pakistan. Leveraging data from Pakistan’s latest Demographic and Health Survey (2017–18), the study employed: (1) the Composite Index of Anthropometric Failure (CIAF) to comprehensively measure overlapping malnutrition dimensions; (2) a decomposition analysis of the Concentration Index to quantify wealth- and region-based inequalities; and (3) the Oaxaca-Blinder decomposition to isolate structural determinants of nutritional disparities. The results uncover the underlying mechanisms driving inequality, offering policymakers empirically grounded strategies to mitigate Pakistan’s entrenched intergenerational malnutrition crisis.

## 2 Data and methodology

### 2.1 Theoretical foundation: cardinal health measurement approach for inequality in child malnutrition, and study variables

The cardinal approach rooted in microeconomic theory is widely employed to estimate concentration indices that capture income- or wealth-related inequalities in health outcomes (26). A central methodological challenge in this domain arises from the fact that health data are frequently available only at the ordinal level. Many health indicators in population-based surveys are inherently categorical or ordinal, such as the commonly used self-assessed health question, ‘How would you rate your overall health?’ This question typically offers response options ranging from ‘excellent’ or ‘very good’ to ‘poor’ or ‘very poor.’ Despite its simplicity, this measure has consistently been validated as a reliable proxy for general health status (27). Nevertheless, certain health metrics—such as Body Mass Index (BMI) or child’s anthropometric measurements, such as HAZ, WAZ, and WHZ z-scores on a scale from 0 to 1—are cardinal in nature and more directly amenable to standard inequality measures (28). In addressing health inequalities, researchers may either apply inequality measures suited to ordinal data or convert ordinal variables into cardinal equivalents to facilitate the use of conventional concentration indices (29).

In the assessment of health inequalities using measures such as the concentration index, it is common practice to dichotomize health outcomes—e.g., into healthy versus non-healthy categories. Health variables must be continuous or binary in order to be used with the concentration index and its associated slope index of inequality (SII), allowing them to be interpreted within a cardinal framework, often on a latent utility scale ranging from 0 to 1 (30–32). This approach builds on the concept of ‘response category cut-point shift,’ which adjusts for systematic variations in reporting across socioeconomic groups. Wagstaff and Van Doorslaer, advanced this methodology by proposing a latent ill-health variable assumed to follow a log-normal distribution, from which ordinal health responses are generated—thus avoiding the use of arbitrary mid-point values and instead relying on estimated cut-points (33). More recently, a study demonstrated that when ordinal health data at the individual level are available, carnivalization techniques can be effectively employed to measure health inequality, thereby enabling the application of standard inequality metrics within population health research (34).

The major anthropometric variables used to quantify child undernutrition in this study are height-for-age (HAZ), weight-for-age (WAZ), and weight-for-height (WHZ) z-scores. These indices provide crucial insights into early childhood growth and are vital for assessing the prevalence and severity of undernutrition. Additionally, these indicators are used to construct the Composite Index of Anthropometric Failure (CIAF), based on the World Health Organization’s (2009) classification guidelines (35). The CIAF has a complete system for finding different types of undernutrition. It divides children into seven groups that cannot be joined together: (1) no failure, (2) stunted only, (3) wasted only, (4) underweight only, (5) stunted and underweight, (6) wasted and underweight, and (7) stunted, wasted, and underweight. For analytical purposes, the prevalence of undernutrition is estimated by aggregating all categories of anthropometric failure, excluding the “no failure” group. A binary outcome variable is then constructed, coded as “1” for children experiencing any form of anthropometric failure (i.e., malnourished) and “0” for those without any failure, enabling the application of dichotomous health inequality measures.

In this study, the Wealth Index—categorized into quintiles (poorest, poorer, middle, richer, richest)—and region of residence (Punjab, Sindh, Khyber Pakhtunkhwa, Balochistan, Azad Jammu and Kashmir, Federally Administered Tribal Areas, and Islamabad Capital Territory) are included as primary explanatory variables. Included as important control variables are the following: the child’s age (a continuous variable), the mother’s education level (none, elementary, secondary, or higher), and the child’s place of residence (urban or rural). These categories help to account for potential confounding factors. We chose these factors because they have been shown to be important in studies on socioeconomic determinants of health and child undernutrition.

### 2.2 Data and description

The Pakistan Demographic and Health Survey (PDHS) was conducted in 2017 and 2018 and included 4,499 children under the age of five. The survey is representative of the country’s population and is meant to offer accurate estimates of health, nutrition, and population size. Data collection was place from November 22, 2017, to April 30, 2018, and was carried out by the National Institute of Population Studies (NIPS) in partnership with Pakistan’s Ministry of National Health Services, Regulations, and Coordination. Enumeration blocks (EBs) from the Pakistan Population and Housing Census of 2017 formed the basis of the sampling frame. First, 580 enumeration areas (EAs) were chosen using a two-stage stratified random sampling method. Then, 28 houses were randomly selected for each EA. Anthropometric measures were obtained from around one-third of the 16,240 households that provided data. Important nutritional indicators such as height-for-age (HAZ), weight-for-age (WAZ), and weight-for-height (WHZ) z-scores were calculated using data on children’s weight, age, and height from 0 to 59 months. The sample provides accurate estimates on a national and provincial scale, and it accurately represents both urban and rural populations.

To ensure the representativeness of our estimates, we accounted for the complex sampling design of the PDHS 2017–18 in all analyses. The survey employs a stratified, multi-stage cluster sampling approach, requiring appropriate weighting to address unequal selection probabilities and produce nationally valid results. We applied the PDHS-provided sampling weights (v005/1,000,000) to both descriptive statistics and regression models. The statistical analysis was explicitly incorporated: (1) Primary sampling units (v021) to adjust for cluster-level correlations; (2) Stratification variables (v022) to account for regional sampling frames; (3) Normalized household weights (v005) to correct for differential selection probabilities. Validation checks confirmed that our weighted estimates aligned precisely with official PDHS benchmarks, including the 38% stunting prevalence among children under five. This rigorous weighting approach guarantees that our findings accurately reflect population-level patterns of undernutrition across Pakistan’s diverse socioeconomic and geographic strata.

### 2.3 Models for statistical analysis

#### 2.3.1 Concentration index and decomposition

In order to capture the degree of wealth or socioeconomic inequality in nutritional outcome of under-five children in Pakistan, this study used the Concentration Index (CI) which is recommended by Kakwani et al. (36) and Wagstaff (37). The formula for CI is as follow:


(1) $$C=\frac{2}{n.u}[\sum_{i=1}^{n}Y_{i}\;R_{i}]-1$$

Where ″″u″ is the sample size which is the mean of child nutritional outcome, the value of child nutrition denoted by $\prime \prime \prime \prime {y_{i}}^{\prime \prime \prime \prime }$ in $\prime \prime \prime \prime \mathrm{it}h^{\prime \prime \prime \prime }$ person, the rank of wealth status of child is denoted by “Ri.” “The concentration index (CI) ranges from −1 to +1, with a value of zero indicating the absence of socioeconomic status (SES)-related inequality (36, 38).

To assess the contribution of socioeconomic factors to inequality in childhood malnutrition, the Concentration Index was decomposed using a regression-based approach (39). The linear specification allows the outcome variable ″″y″ to be expressed as a function of ″″k″ socioeconomic determinants, enabling quantification of each factor’s contribution to overall inequality.


(2) $$y_{i}=a+\sum_{k}\beta_{k}x_{k}+\epsilon$$

In this model, $\beta_{k}$ denotes the coefficient for each independent variable, and ϵ represents the error term. By substituting $y_{i}$ from the regression equation into the expression for the Concentration Index, we obtain a decomposition that attributes inequality in the outcome variable to the contributions of its explanatory factors.


(3) $$c=\sum_{k}(\frac{\;\beta_{k}\;{\bar{x}}_{k}}{\mu })c_{k}+\frac{\;{\mathrm{CG}}_{\epsilon }}{\mu }=c_{\hat{y}}+\frac{\;{\mathrm{CG}}_{\epsilon }}{\mu }$$

In this decomposition, ${\bar{x}}_{k}$ represents the mean of the explanatory variable $x_{k}$ , $c_{k}$ denotes the Concentration Index of $x_{k}$ and ${\mathrm{CG}}_{\epsilon }$ indicates the generalized Concentration Index of the error term (ϵ) . The term $c_{k}$ quantifies the degree to which each determinant is unevenly distributed across socioeconomic groups, capturing its contribution to malnutrition inequality. The residual component $\frac{\;{\mathrm{CG}}_{\epsilon }}{\mu }$ , reflects the portion of inequality that remains unexplained by the included determinants, representing the residual variation across socioeconomic strata.

This study employs the Wagstaff et al. decomposition method adapted for binary outcomes through the Erreygers Corrected Concentration Index (ECI) (39). This approach disaggregates inequality in CIAF into contributions from individual determinants and a residual component. Critical socioeconomic factors—such as the child’s age and sex, maternal education, residential area, region, and wealth status—were incorporated as independent variables in a logistic regression model. The influence of these variables on socioeconomic disparities in malnutrition has been well established in prior research (40–42).

#### 2.3.2 Oaxaca-Blinder decomposition

This study utilizes the Oaxaca-Blinder decomposition to disentangle the sources of inequality between groups (43, 44). The method separates the difference in the outcome variable into explained and unexplained components. The explained component accounts for differences attributable to observed characteristics between the most and least advantaged groups, whereas the unexplained component reflects disparities driven by unmeasured factors beyond the scope of this analysis.


(4) ΔY_=(X_A+X_B)′β^R︸Explainedunexplained+X_A′(β^A−β^B)︸unexplainedA+X_B′(β^R−β^B)︸unexplainedB

Let assume ${}^{\prime \prime \prime \prime }Y^{\prime \prime \prime \prime }$ represents the outcome variable that quantifies inequality of opportunity in poor nutritional status. The analysis compares two groups: Group A, comprising the poorest and poorer wealth quintiles (coded 0), and Group B, including the richer and richest quintiles (coded 1). The variation in ${}^{\prime \prime \prime \prime }Y^{\prime \prime \prime \prime }$ is modeled as a function of a vector of determinants ${}^{\prime \prime \prime \prime }X^{\prime \prime \prime \prime }$ within each group using a linear analysis framework, expressed as follows:


(5) $$\mathrm{Io}P_{\text{Mean}}^{R}=\beta_{\text{Mean}}^{R}+\sum_{j=1}^{J}.\beta_{j}^{R}X_{j\;\text{mean}}^{R}$$

(6) $$\mathrm{Io}P_{\text{Mean}}^{U}=\beta_{\text{Mean}}^{U}+\sum_{j=1}^{J}.\beta_{j}^{U}X_{j\;\text{mean}}^{U}$$

In this framework, the superscripts ${}^{\prime \prime \prime \prime }R^{\prime \prime \prime \prime }$ and ${}^{\prime \prime \prime \prime }U^{\prime \prime \prime \prime }$ represent the rich and poor groups, respectively, while ${}^{\prime \prime \prime \prime }X^{\prime \prime \prime \prime }$ denotes a set of ${}^{\prime \prime \prime \prime }J^{\prime \prime \prime \prime }$ measured predictors. Inequality of opportunity LoP is reflected by the outcome variable, with $\mathrm{Lo}P_{\text{mean}}$ indicating its average value. The coefficients reflect the strength of association between each predictor $X_{j}$ and LoP . The overall difference between groups can thus be decomposed as follows:


(7) $$\mathrm{Io}P_{\text{Mean}}^{R}-\mathrm{Io}P_{\text{Mean}}^{U}=(\beta_{0}^{R}-\beta_{0}^{U})$$

After straightforward algebraic manipulation, the Oaxaca-Blinder Decomposition can be formally uttered as follows:


(8) $$\begin{array}{l} \mathrm{Io}P_{\text{Mean}}^{R}-\mathrm{Io}P_{\text{Mean}}^{U}=[\sum_{j=1}^{J}(X_{j\;\text{mean}}^{R}-X_{j\;\text{mean}}^{U})\beta_{j}^{R}]+\\ {}[(\beta_{0}^{R}-\beta_{0}^{U})+\sum_{j=1}^{J}(\beta_{j}^{R}-\beta_{j}^{U})X_{j,\text{mean}}^{R}] \end{array}$$

The first term on the left-hand side captures the ‘explained’ portion of inequality, reflecting differences between the poor and rich groups attributable to observed factors. The second term represents the ‘unexplained’ component, accounting for disparities arising from variations in coefficients and mean values of these factors.

## 3 Findings of the study

### 3.1 Descriptive statistics

The analysis starts with distribution of anthropometric data. Anthropometric assessment serves quantifying malnutrition prevalence within populations and subgroups. This foundational analysis enables: (A) Comparative assessment against healthy reference populations; (B) Identification of distinct malnutrition subtypes (stunting, wasting, underweight); (C) Preliminary characterization of population nutritional heterogeneity. The distributional patterns provide critical insights for subsequent etiological investigation of malnutrition patterns.

**Figure 1:**
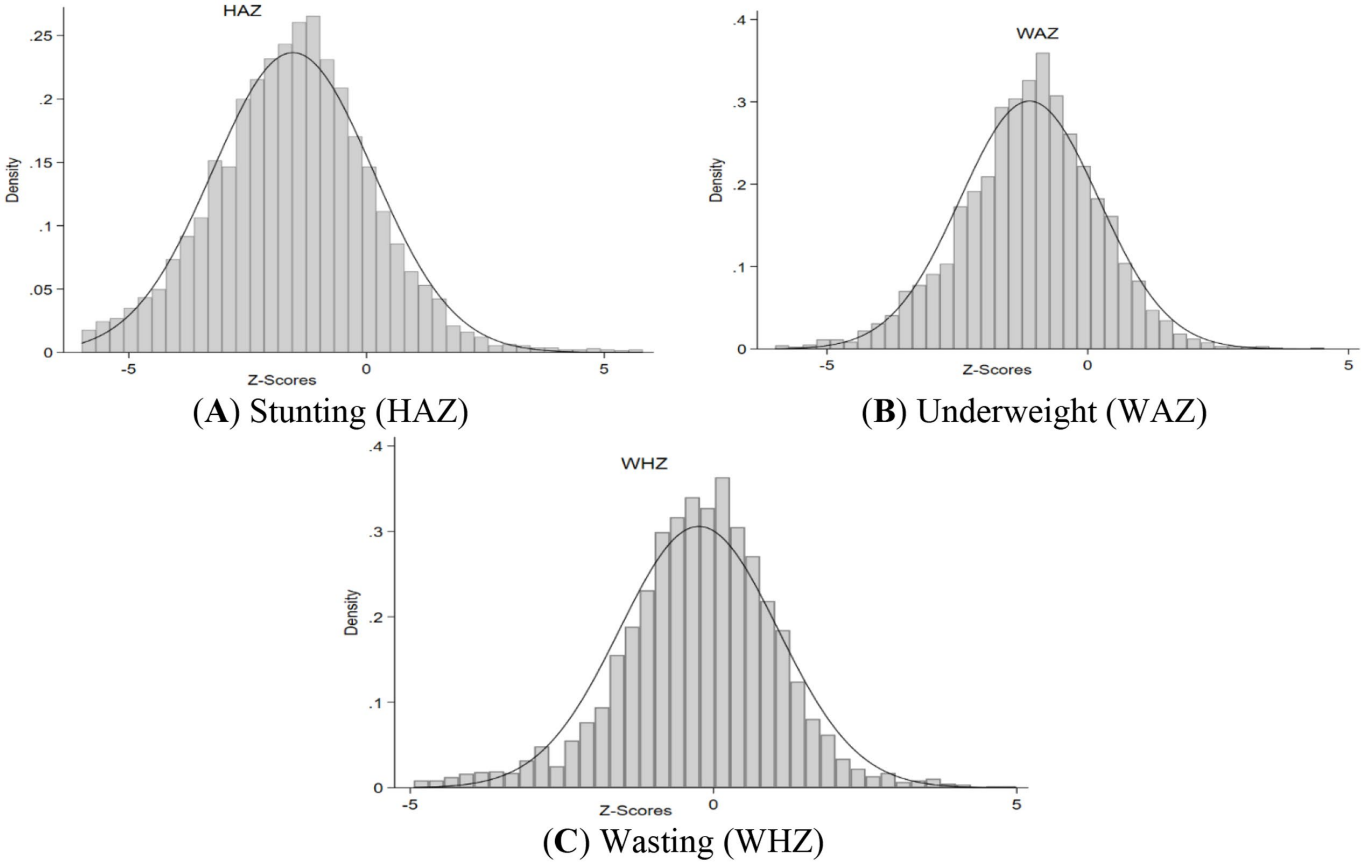
Distribution of *Z*-scores in PDHS 2017–18. **(A–C)** depicted the defects in z-scores in HAZ, WAZ, and WHZ in data.

Figure 1 depicted the defects in z-scores in HAZ, WAZ, and WHZ in data. Similarly, this study begins by examining bivariate relationships between anthropometric indicators and socioeconomic characteristics through graphical analysis. While these descriptive visualizations provide valuable initial insights into potential associations, we intentionally avoid making causal inferences at this exploratory stage.

**Figure 2:**
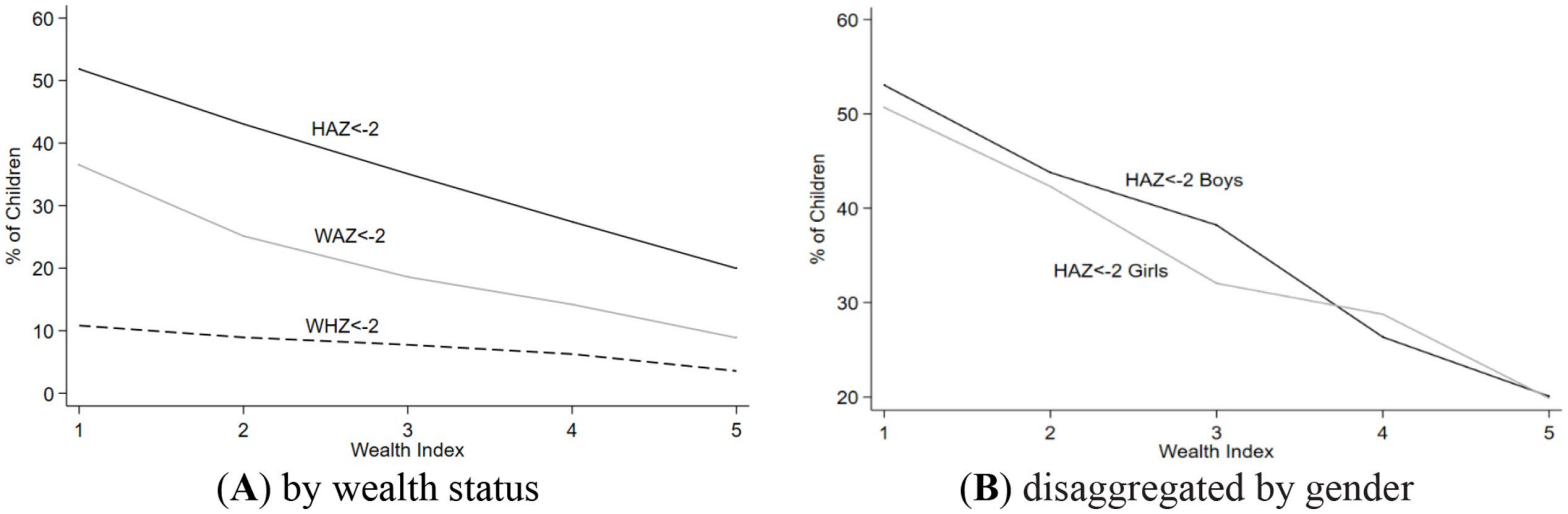
Nutritional outcome for different wealth status quintiles, and a disaggregation by sex. **(A)** It represents stunting, wasting, and underweight prevlance among children by wealth status. While **(B)** represents the stunting, wasting, and underweight prevlance among children by wealth status, disaggregated by child gender.

Figures 2A,B illustrate the prevalence rates of underweight, stunting, and wasting across different wealth index quintiles in Pakistan. The graphs 2A depicts the expected trend of malnutrition decreasing with an increase in wealth status. Furthermore, the graph 2B shows this expected trend in both girls and boys, emphasizing the inverse relationship between malnutrition and wealth status.

Table 1 estimates malnutrition prevalence to determine how predictor modifications affect the CIAF. Malnutrition is slightly higher in boys (22.48%). The 25–36- and 37-48-month age groups have 10.33% prevalence. Children living in urban areas (17%) experience better nutritional outcomes compared to rural residents (26%). The prevalence is higher in Punjab (7.24%), Sindh (10.21%), KPK (7.02%), and Balochistan (7.46%) children. Mothers with illiteracy have 27.8% greater malnutrition rates. Child malnutrition is higher in poor (13.42%) and poorer (12.39%) households.

**Table 1:** Prevalence of child undernutrition by socio-economic profile (*n* = 4,499).

Variable	Category	Frequency	Percentage	*p*-value
Gender of Child	Girl child	886	21.67	0.67
	Boy child	922	22.48	
Children-age calculated months	0 to twelve months	283	6.93	<0.001
	Thirteen to twenty-four	327	7.95	
	Twenty-five thirty-six	424	10.34	
	Thirty-seven to forty-eight	427	10.32	
	Forty-eight to sixty	348	8.43	
Place of Residence	Rural living	1,075	26.32	<0.001
	Urban living	733	17.81	
Region/Province	Punjab (province)	296	7.24	<0.001
	Sindh (province)	417	10.21	
	KPK (province)	287	7.02	
	Balochistan (province)	307	7.46	
	FATA (province)	188	4.63	
	Gilgit Baltistan (province)	107	2.65	
	Islamabad-Capital	64	1.55	
	Azad Jammu and Kashmir	141	3.48	
Mother’s Education Level	Illiterate	1,146	27.8	<0.001
	0-to-five-year schooling	233	5.70	
	Six-to-twelve schooling	291	7.07	
	Higher studies	137	3.38	
Household Wealth Index/Status	Poorest people class	551	13.42	<0.001
	Poorer people class	508	12.39	
	Middle people class	323	7.90	
	Richer people class	255	6.21	
	Richest people class	172	4.23	

Authors’ estimation. Statistical significance assessed at the 1 and 5% levels. Table 1 depicts cross-tabulation results only for malnourished children category.

### 3.2 Outcome of concentration index

To assess inequality across subgroups, relative measures are often employed. While simple ratios between two groups provide a basic comparison, more comprehensive analysis requires measures that account for multiple groups simultaneously. The Concentration Index (CIX), conceptually related to the Gini coefficient, serves this purpose by quantifying how an outcome variable is distributed across a ranked socioeconomic spectrum—typically from the most disadvantaged (poorest) to the most advantaged (richest).

**Figure 3:**
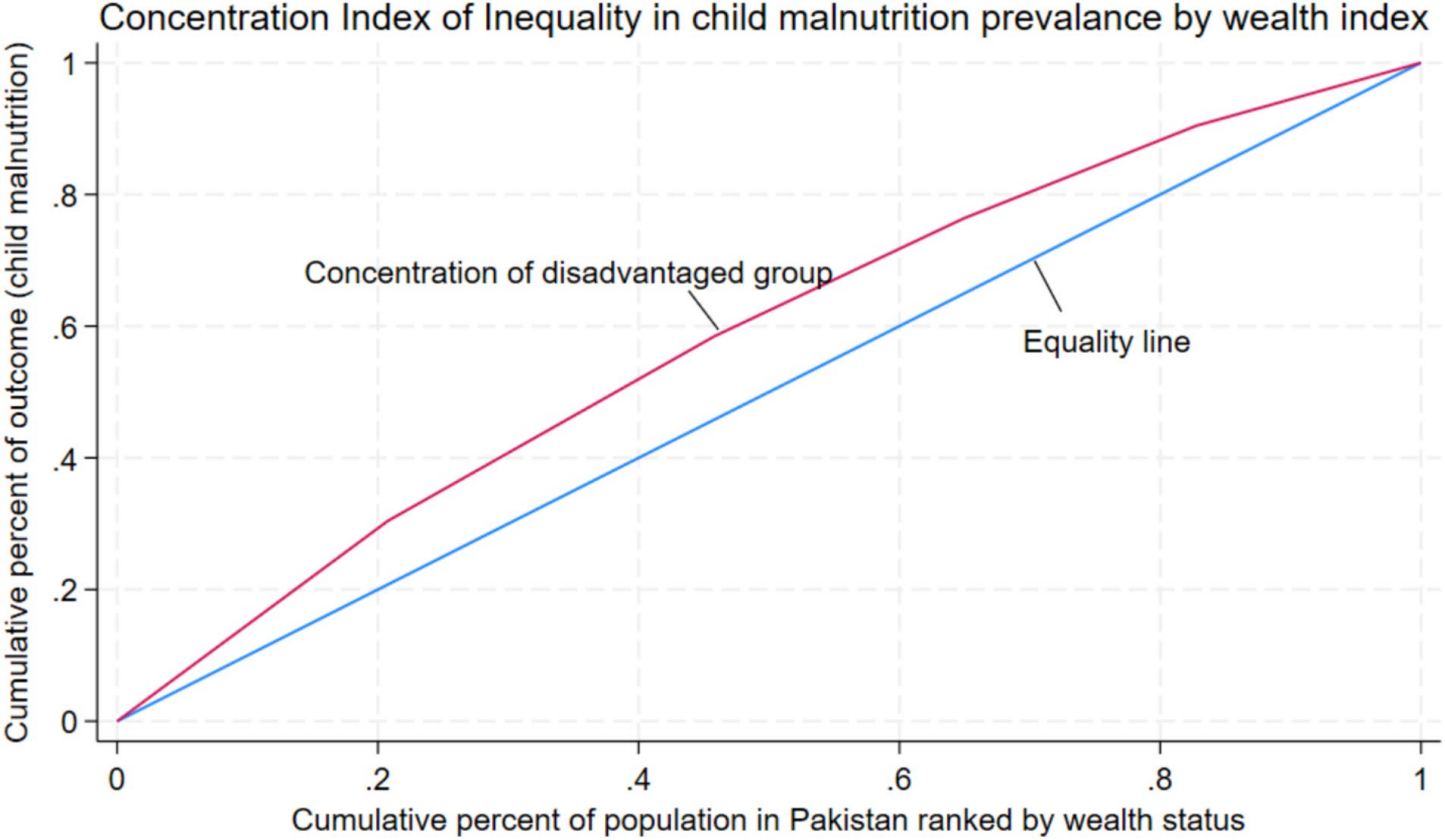
Concentration index for inequalities in child malnutrition prevalence by wealth status.

In Figure 3, wealth status serves as the ranking variable, with the lowest quintiles representing the most disadvantaged (poorest) and the highest quintiles the most advantaged (richest). The x-axis displays the ranking variable, while the y-axis represents the outcome. The 45-degree line denotes perfect equality, indicating an even distribution of the outcome across all groups. A concentration curve below this line signifies that the outcome is concentrated among the advantaged, whereas a curve above indicates concentration among the disadvantaged. The Concentration Index (CIX), which ranges from −1 to +1, serves as a measure of inequality, quantifies this inequality: positive values reflect concentration among the affluent, negative values among the poor, and zero denotes perfect equality. The Concentration Index value of −0.154 (*p* < 0.001) indicates a significant pro-poor inequality in child malnutrition, with the burden disproportionately concentrated among the poorest wealth quintiles. The disparity in malnutrition prevalence between the richest and poorest groups reaches 65.6%. In this analysis, wealth status is represented on the x-axis, with child malnutrition plotted on the y-axis. As depicted in Figure 3, the above the line of equality, the concentration curve remains constant, indicating that child malnutrition disproportionately affects the most disadvantaged groups.

By taking the complete distribution of the population into account, the Slope Index of Inequality (SII) offers another way to evaluate absolute inequality. It ranges from −1 to +1 and measures the difference in the outcome variable between the top and lowest socioeconomic categories. Perfect equality is represented with a value of zero, while positive values suggest that the outcome is more concentrated among the wealthier segments, and negative values indicate a greater burden on the poorer or most disadvantaged groups.

**Figure 4:**
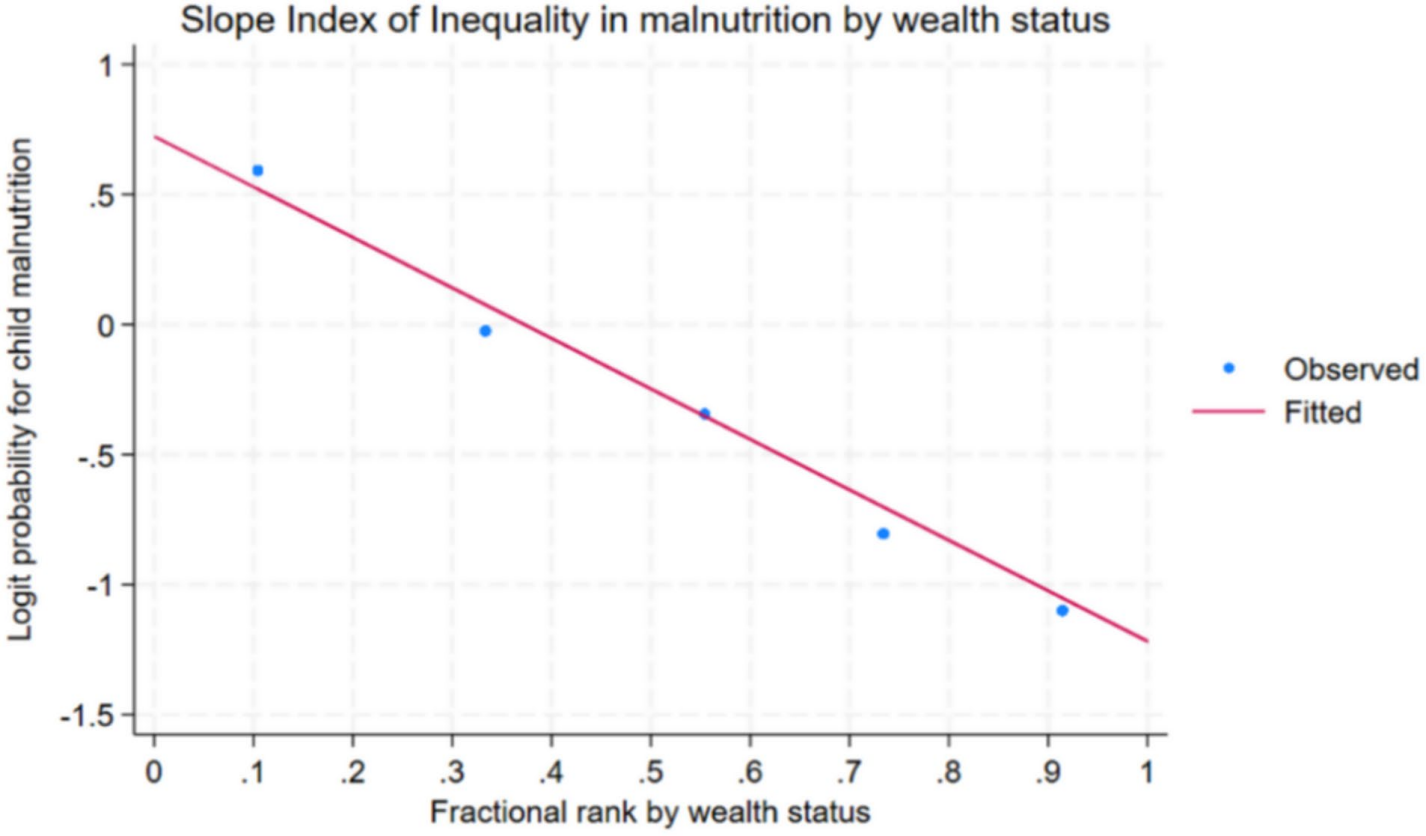
Slop index of inequality (SII) in child malnutrition prevalence by wealth status.

In Figure 4, each dot represents the rate of child malnutrition within a particular wealth quintile, while the fitted line illustrates the overall trend across the socioeconomic gradient. The downward slope of the line indicates a decline in malnutrition with increasing wealth, yielding a negative Slope Index of Inequality (SII) value of −0.284. This negative SII confirms that child malnutrition is disproportionately concentrated among the poorest groups, with significantly lower prevalence observed in the wealthiest quintile.

The relative Concentration Index (−0.0202) and absolute value (−0.0168) for regions indicate that child undernutrition is disproportionately concentrated in Pakistan’s more deprived regions. The malnutrition prevalence gap between the most deprived and most developed regions is 14.2%. To enhance the visualization of regional disparities, the study employs an equiplot, which effectively illustrates and compares absolute inequalities across provinces. This analysis further explores how wealth-related disparities in child malnutrition vary across different regions of Pakistan.

**Figure 5:**
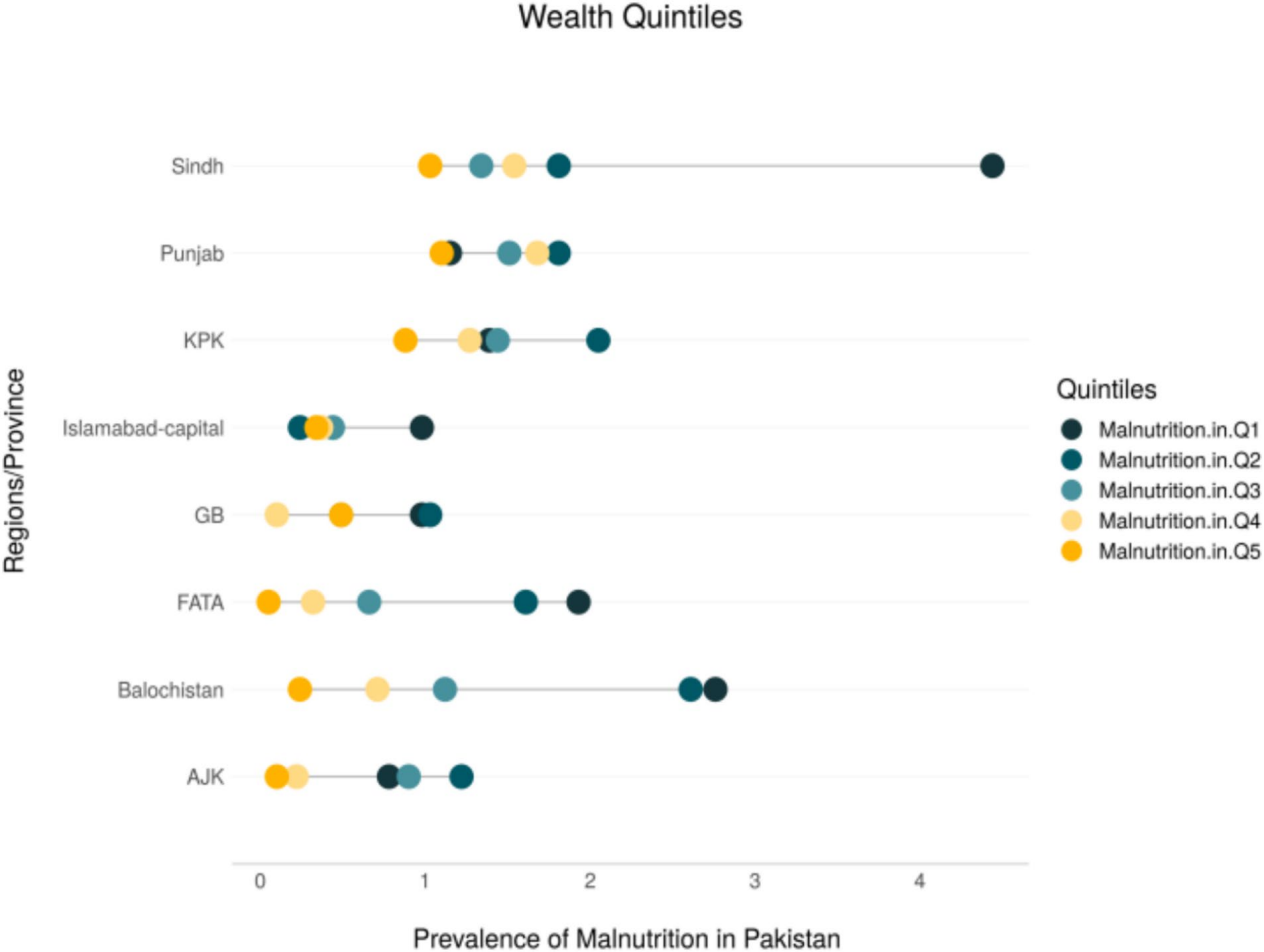
Inequalities in child malnutrition prevalence by wealth status across region/provinces.

Figure 5 presents an equiplot with malnutrition prevalence on the x-axis and regions/provinces on the y-axis. Each dot corresponds to a wealth quintile, with darker dots representing the poorest groups and lighter (yellow) dots indicating the wealthiest. The length of the line connecting these dots reflects the degree of absolute inequality within each region. The plot reveals pronounced disparities in malnutrition between the poorest and wealthiest groups in Sindh, Balochistan, Khyber Pakhtunkhwa (KPK), and FATA—regions identified as among the most underdeveloped in Pakistan.

Moreover, below Table 2 depicts the more detailed presentation of presents the Concentration Index values (CI) and Table 3 shows the decomposition results (elasticities and contributions).

**Table 2:** Concentration indices (CI) for wealth and region-based inequality in undernutrition.

Variables	Concentration index (C_k_)-CI	*p*-values
Wealth quantiles		
Poorest	−0.792	<0.001
Poorer	−0.611	<0.001
Middle	−0.399	<0.001
Rich	−0.208	0.005
Overall (Rich-Poor gap)	−0.154	<0.001
Regions		
Sindh	−0.124	<0.001
KPK	−0.183	<0.001
Balochistan	−0.316	<0.001
FATA	−0.402	<0.001
AJK	0.113	0.012
ICT	0.299	0.006
Overall (Regional gap)	−0.0202	0.008
Other characteristics		
Rural (vs Urban)	−0.227	<0.001
No Education (vs Higher)	−0.653	<0.001

Source: Author estimations. Concentration index (CI) = Concentration Index of each determinant (how unequally it’s distributed).

**Table 3:** Decomposition analysis for inequality in undernutrition.

Variables	Elasticity (η_k_)	Absolute contribution (η_k_C_k_)-SSI	Percentage contribution	*p*-values
Poorest	0.137	−0.109	31.5	<0.001
Poorer	0.113	−0.069	19.9	<0.001
Overall, wealth contribution between rich and poor	–	**0.284** (Erreygers CI value)	**65.6**	**<0.001**
Balochistan	0.049	−0.016	4.5	<0.001
Overall, region contribution between developed and underdeveloped	–	**−0.0168** (Erreygers CI value)	**14.2**	**0.008**
No Education	0.128	−0.084	24.1	<0.001
Residual	–	−0.014	3.8	–
Total	–	**0.345**	**100**	<0.001

Source: Author estimations. Elasticity **=** coefficient × mean of variable / mean of CIAF. Contribution **=** Elasticity × CI. Positive contribution means pro-rich inequality; negative indicates a pro-poor pattern. Elasticity measures the responsiveness of undernutrition to changes in a determinant. It was computed as: $\eta_{k}=\beta_{k}\frac{\text{Mean of}\;X_{k}\;}{\text{Mean of Undernutrition}\;(\text{CIAF})}$ Where: $\beta_{k}$ Regression coefficient of variable $X_{k}$ (from the logistic model). Mean of X_k_ = Average value of the independent variable (e.g., proportion of children in the “poorest” wealth quantile). A higher absolute value of η_k_ indicates a stronger influence of that variable on undernutrition inequality. The sign of η_k_ shows the direction of the relationship (e.g., negative elasticity for “poorest” wealth quantile means undernutrition decreases as wealth increases). Suppose for “Poorest wealth quantile” in Table 2: β_k_ (coefficient) = 0.45 (from logistic regression); Mean of X_k_ = 30% of children are in the “poorest” category; Mean of CIAF = 20% undernutrition prevalence: $\eta_{k}=0.45\frac{0.30\;}{0.20}=0.137$ (matching with Table 3). Bold values are CI results, especially overall difference between rich and poor, bolded for visibility.

The concentration index analysis in Table 2 reveals profound socioeconomic and geographic disparities in child undernutrition across Pakistan. Wealth-based inequalities demonstrate particularly striking patterns, with the poorest households (CI = −0.792, *p* < 0.001) experiencing nearly four times greater malnutrition concentration than the rich (CI = −0.208, *p* = 0.005). The overall rich-poor gap (CI = −0.154, *p* < 0.001) confirms the systematic disadvantage faced by economically marginalized populations.

Regional disparities show equally concerning patterns, with FATA (CI = −0.402, *p* < 0.001) and Balochistan (CI = −0.316, *p* < 0.001) exhibiting the most severe concentration of undernutrition. Notably, these conflict-affected regions show significantly worse outcomes than more developed areas like ICT (CI = 0.299, *p* = 0.006) and AJK (CI = 0.113, *p* = 0.012). The overall regional gap (CI = −0.0202, *p* = 0.008) highlights the persistent geographic inequities in nutritional outcomes.

Additional determinants reveal important patterns: (A) Rural children face substantially higher undernutrition concentration than urban peers (CI = −0.227, *p* < 0.001); (B) Maternal education shows the strongest single association, with uneducated mothers’ children having dramatically worse outcomes (CI = −0.653, *p* < 0.001) compared to those with educated mothers.

These results collectively demonstrate that child undernutrition in Pakistan follows clear socioeconomic gradients, with wealth status, geographic location, and maternal education serving as primary structural determinants of nutritional inequality.

The decomposition analysis in Table 3 reveals striking patterns in the structural determinants of child undernutrition inequality (Table 3). Wealth disparities emerge as the predominant driver, accounting for 65.6% of total inequality (Erreygers CI = 0.284, *p* < 0.001). Within this wealth effect, the poorest households contribute nearly one-third of total inequality (31.5%, absolute contribution = −0.109, *p* < 0.001), while the poorer quintile adds another 19.9% (−0.069, *p* < 0.001). These findings demonstrate the profound nutritional penalty faced by economically marginalized populations.

Maternal education represents the second largest contributor, explaining 24.1% of inequality (−0.084, *p* < 0.001). The magnitude of this effect highlights how intergenerational educational disadvantages translate into nutritional deprivation. Regional disparities, while smaller in comparison, remain statistically significant, with underdeveloped regions contributing 14.2% to overall inequality (Erreygers CI = −0.0168, *p* = 0.008). Balochistan alone accounts for 4.5% of the total inequality (−0.016, *p* < 0.001), underscoring the geographic dimension of nutritional injustice.

The comprehensive model explains 96.2% of observable inequality (residual = 3.8%), with the total concentration index reaching 0.345 (*p* < 0.001). This decomposition makes clear that Pakistan’s child undernutrition crisis is fundamentally structural in nature, with wealth status, maternal education, and regional development gaps collectively responsible for the vast majority of nutritional disparities.

### 3.3 Findings of Oaxaca-Blinder decomposition

To uncover the underlying drivers of wealth and region-based disparities in child undernutrition, Table 4 employs the Oaxaca–Blinder decomposition, disentangling how differences in characteristics and structural factors jointly shape the inequality in CIAF outcomes.

**Table 4:** Oaxaca-Blinder decomposition results for inequality in undernutrition.

Components	Measures	Value	*p*-value
1. Predicted CIAF Rates	Poorest (group_1/A)	57.9%	–
	Richest (group_2/B)	32.5%	–
	Total Gap	25.5% points	<0.001
2. Decomposition Summary	Measures	Coefficient	% of Total Gap (p-value)
	Endowments (Explained)	0.166	65.1% (<0.0001)
	Coefficients (Unexplained)	0.117	45.9% (0.007)
	Interaction Term	−0.028	−11% (0.535)
3. Variable-Level Contributions – Endowments (Explained)	Measures	Coefficient	p-value
	Mother’s Education (mel_1: Low/Illiterate)	+0.153	<0.0001
	Region (Punjab & KPK)	+0.018	0.157
	Other Variables (e.g., Region_1, Child Age, Residence Place)	Small/Insignificant	–
4. Variable-Level Contributions – Coefficients (Unexplained)	Measures	Coefficient	*p*-value
	Region-1 (Sindh & Balochistan)	−0.077	0.015
	Mother’s Education (Low/Illiterate)	−0.044	0.067
	Place of Residence (TPR_1: Rural)	−0.073	0.056

Source: Author’s estimation.

The result of Oaxaca-Blinder decomposition is given in Table 4. The Oaxaca-Blinder decomposition reveals a substantial wealth-based disparity in child undernutrition in Pakistan. Children in the poorest households show a 25.5 percentage-point higher malnutrition prevalence than their richest counterparts (*p* < 0.001). About 65% of this inequality is attributable to explained differences (endowments) in characteristics such as maternal education and regional/province location (*p* < 0.0001). Notably, maternal education alone accounts for over 60% of the explained gap. Among the endowment factors, maternal education emerges as the most influential driver of inequality. The prevalence of low or no education among mothers in poorer households contributes 15.3 percentage points to the explained gap (*p* < 0.0001), underscoring its pivotal role in nutritional disparities.

The remaining 46% is unexplained, highlighting potential structural inequities. Regional/provincial effects are particularly important, suggesting that beyond individual-level disparities, geographic inequality significantly contributes to child undernutrition in Pakistan. In the unexplained component, structural disparities are more pronounced. Regional effects, particularly in Sindh and Balochistan, show a significant negative coefficient (−0.077; *p* = 0.015), indicating that children in these provinces face significant disadvantages in nutritional outcomes. Similarly, living in rural areas (−0.073; *p* = 0.056) has a negative effect, meaning that children from poor households benefit less from their place of residence compared to those in wealthier, urban settings.

## 4 Discussion

This study provides a comprehensive examination on wealth and regional-based disparities in child undernutrition in Pakistan, leveraging nationally representative data from the PDHS 2017–18. By employing both the Concentration Index with decomposition analysis along with Oaxaca-Blinder decomposition. The findings offer robust evidence that child malnutrition is not merely a health issue but a manifestation of deep-rooted structural inequalities.

The results demonstrate an unadorned pro-poor disparity in child undernutrition, as evidenced by negative values of both the relative and absolute concentration indices. Poverty or poor wealth position regularly emerges as the primary predictor of child malnutrition, which is consistent with broader literature from other low- and middle-income countries (45–48). In this study, household wealth alone accounted for 45.6% of inequality in child nutritional outcomes, affirming that economic deprivation remains a central barrier to adequate nutrition. In most of the under developing countries such Pakistan, Bangladesh, India, Nepal (South Asia), and Ethiopia the wealth-related breaches in child malnutrition were increasingly high (49–51). These findings supported by literature highlight the urgency of incorporating equity-sensitive frameworks into national nutrition and poverty alleviation strategies.

Furthermore, the Oaxaca-Blinder decomposition underscores a pronounced wealth gap in malnutrition prevalence, with children from poor households facing a 25.5% points higher likelihood of undernutrition. Importantly, approximately two-thirds of this gap is explained by differences in household wealth, maternal education, and regional location, indicating that these structural determinants account for the majority of observed disparities. This reinforces prior evidence suggesting that improving socioeconomic conditions, particularly through maternal empowerment and regional development, is critical to tackling malnutrition at larger scale (52, 53).

The study also brings attention to geographic inequities in nutritional outcomes. Provinces such as Sindh, Khyber Pakhtunkhwa, Balochistan, and FATA collectively contribute 12.9% of inequality, revealing substantial spatial disparities. Additionally, negative coefficients produced by Oaxaca-Blinder, indicates that children in these provinces face significant disadvantages in nutritional outcomes. Similarly, living in rural areas has a negative effect and contributes to 6.5% of inequality in child malnutrition, meaning that children from poor households benefit less from their place of residence compared to those in wealthier, urban settings. These findings corroborate earlier research that links regional underdevelopment and inadequate service delivery with poor child nutrition, and health outcomes (54–57). Another study from middle-east found a significant regional wealth-based inequalities in stunting prevalence among Egypt, Jordan, and Yemen (58). Addressing these disparities necessitates not only targeted provincial interventions but also systemic reforms in resource allocation and governance structures.

Another salient finding is the significant role of maternal no education, which accounts for 24.1% of nutritional inequality. Maternal literacy has long been associated with improved child health and nutrition through pathways such as increased autonomy in household decision-making (59–61). Thus, investing in girls’ education represents a transformative policy lever for improving intergenerational health outcomes.

In general, these results ccollectively found that structural disadvantages related to socioeconomic status and geographic location**—**especially in deprived provinces, and among the lowest wealth quintiles—substantially drive nutritional inequality among Pakistani children. This indicates that child malnutrition in Pakistan is comparatively lower about remote nutritional deficiencies and more about enduring socioeconomic, structural, and institutional drawbacks. Tackling undernutrition, therefore, requires a multisectoral approach that addresses the root causes of inequality. Nevertheless, the study makes a compelling case for reorienting nutrition policy in Pakistan toward structural determinants of health. By foregrounding the roles of wealth, education, and geography, it provides actionable insights for policymakers aiming to disrupt the intergenerational transmission of undernutrition and inequality.

## 5 Conclusion

The objective of this study is to assess the wealth and regional-based inequalities in child undernutrition in Pakistan, using latest available nationally representative health data (PDHS 2017–18). Our findings reveal that Pakistan’s child undernutrition crisis stems from entrenched structural inequities, with wealth disparities (45.6%), maternal education gaps (24.1%), and regional marginalization (14.2%) collectively explaining 65% of nutritional inequalities. The 25.5 percentage point malnutrition gap between poor and wealthy households underscores the urgent need for equity-focused policy reforms. These results demand a fundamental shift from conventional nutrition interventions toward multisectoral strategies addressing root causes. Three priority action areas emerge: First, economic empowerment through scaled-up, nutrition-sensitive social protection programs targeting ultra-poor households. Second, geographically-precise investments in health infrastructure and food security for high-disparity regions like Balochistan and FATA. Third, accelerated girls’ secondary education initiatives coupled with nutrition literacy components. The health system must develop equity monitoring mechanisms to track subnational inequality patterns while fostering cross-sectoral coordination. These structural interventions - political as much as technical - represent Pakistan’s most viable pathway to break intergenerational malnutrition cycles.

## 6 Practical implications

Based on findings this study recommends some practical policy implication which are as follows: (1) *Equity-Centered Nutrition Policies:* The pronounced pro-poor concentration of child undernutrition calls for the redesign of national nutrition strategies to explicitly prioritize the poorest households. Conditional cash transfer programs, targeted food supplementation, and social protection schemes should be expanded to reach the most vulnerable populations. (2) *Targeted Regional Investment:* The significant contribution of regional disparities—particularly in Sindh, Balochistan, Khyber Pakhtunkhwa, and FATA—signals an urgent need for geographically targeted investments. Strengthening health systems and scaling up community-based nutrition programs in these underserved areas are essential to reduce interprovincial gaps. (3) *Empowering Maternal Education:* With maternal illiteracy accounting for nearly a quarter of malnutrition inequality, policies that promote girls’ education and adult female literacy can have substantial downstream effects on child health. Cross-sectoral collaboration between health and education sectors is crucial for designing integrated maternal and child development programs. (4) *Multisectoral Governance Approach:* Since structural factors such as wealth status, education, and geography jointly drive nutritional inequality, isolated health sector interventions will be insufficient. Effective responses must align with broader poverty alleviation, women’s empowerment, and rural development agendas, guided by interministerial coordination and equity-sensitive budget allocation. (5) *Breaking the Intergenerational Cycle of Malnutrition:* Addressing the root causes of undernutrition through economic empowerment and educational advancement equally in all regions is critical not only for current health outcomes but also for preventing the transmission of poverty and malnutrition across generations.

### 6.1 Contribution of this study

This study provides groundbreaking evidence on Pakistan’s child undernutrition crisis by revealing how structural inequalities—particularly wealth disparities (45.6%) and regional marginalization (12.9%)—drive nutritional inequities more profoundly than previously understood. Using novel CIAF-based decomposition methods, we demonstrate that economic factors outweigh even maternal education (24.1%) in shaping outcomes, while exposing unexpected geographic patterns where conflict-affected regions surpass urban–rural divides. Moreover, this is the first comprehensive national-level analysis employing the Composite Index of Anthropometric Failure (CIAF) within an economic measurement framework, as methodologically, we introduced the cardinal health measurement approach, offering a fresh economic perspective on health inequality analysis. Our cardinal measurement approach redefines health inequality analysis, uncovering critical thresholds—like the limited impact of primary education—that demand policy transformation. These findings fundamentally shift the paradigm from individual-focused interventions to systemic solutions prioritizing wealth redistribution, provincial equity, and girls’ secondary schooling to break Pakistan’s malnutrition cycle.

### 6.2 Study limitations and future research directions

This study provides valuable insights into wealth- and region-based inequalities in child undernutrition, yet, certain limitations should be acknowledged: (1) The analysis relies on cross-sectional data from the PDHS 2017–18, which precludes causal inference or temporal assessment. Future research could incorporate longitudinal data to examine inequality trends. (2) The Oaxaca-Blinder decomposition’s unexplained portion may reflect unmeasured factors (e.g., intra-household food distribution or caregiving practices) not captured in DHS surveys. (3) By design, we prioritized wealth and regional disparities to provide an in-depth analysis of structural drivers. While variables like maternal BMI, WASH, and birth order are important, their inclusion would shift focus from our core research question. These factors have been extensively examined in prior studies. Moreover, we have some reasons due to this we have not added other covariates: (A) Maintain analytical precision on these structural determinants; (B) Avoid overcomplicating the decomposition results; (C) Align with our core research question examining macroeconomic/geographic inequities (4) Anthropometric measurements and wealth indices in DHS are subject to measurement error and classification bias, which may influence the precision of the concentration index and decomposition results. (5) Although most common key socioeconomic and demographic variables were included, structural and institutional factors—such as health system performance, policy implementation quality, and nutrition program coverage—could not be incorporated due to study focus only on wealth and region centered indicators. Future researchers can examine the effectiveness of specific intervention packages in reducing these persistent disparities. Moreover, this study conducted on national data (PDHS), for more deeper district-level analysis, the use of province-level data can give insights into localized inequality patterns for the intra-regional disparities. Moreover, future research should evaluate optimal delivery models for equity-centered approaches while assessing their inequality-reducing impacts across wealth and regional strata.

## Data Availability

This study utilized the secondary data of the Pakistan Demographic and Health Survey 2017–18. Data is online freely accessible of DHS program website at: https://dhsprogram.com/data/dataset/Pakistan_Standard-DHS_2017.cfm?flag=1.
